# Os Percentis e Pontos de Corte da Circunferência Abdominal para Obesidade em uma Ampla Amostra de Estudantes de 6 a 10 Anos de Idade do Estado de São Paulo, Brasil

**DOI:** 10.36660/abc.20190043

**Published:** 2020-04-06

**Authors:** José Luiz F Santos, Valentin P. Valério, Rafael N. Fernandes, Ligia Duarte, Antonio C. Assumpção, Jayme Guerreiro, Antonio L. Sickler, Álvaro A. R. Lemos, Jayro G Goulart, Luiz Antonio Machado Cesar, Ibraim Masciarelli Pinto, Carlos Magalhães, Maria Fernanda Hussid, Cleber Camacho, Alvaro Avezum, Carine T. Sangaleti, Fernanda Marciano Consolim-Colombo

**Affiliations:** 1 Sociedade de Cardiologia do Estado de São PauloSão PauloSPBrasilSociedade de Cardiologia do Estado de São Paulo, São Paulo, SP – Brasil; 2 Instituto do CoraçãoSão PauloSPBrasilInstituto do Coração (InCor) – Cardiopneumologia,São Paulo, SP – Brasil; 3 Instituto Dante Pazzanese de CardiologiaSão PauloSPBrasilInstituto Dante Pazzanese de Cardiologia – Cardiologia,São Paulo, SP – Brasil; 4 Universidade Nove de JulhoSão PauloSPBrasilUniversidade Nove de Julho, São Paulo, SP – Brasil; 5 Universidade Estadual do Centro-OesteGuarapuavaPRBrasilUniversidade Estadual do Centro-Oeste - Enfermagem, Guarapuava, PR – Brasil; 6 Universidade de Sao PauloFaculdade de MedicinaHospital das ClínicasSão PauloSPBrasilUniversidade de Sao Paulo Faculdade de Medicina Hospital das Clínicas Instituto do Coração, São Paulo, SP – Brasil

**Keywords:** Criança, Circunferência Abdominal/fisiologia, Obesidade, Estudantes, Parâmetros, Antropometria

## Abstract

**Fundamento:**

A prevalência da obesidade vem aumentando sistematicamente na população, inclusive nas crianças e adolescentes, ao redor do mundo.

**Objetivos:**

Descrever curvas percentílicas de referência para a circunferência abdominal (CA) nas crianças brasileiras e fornecer pontos de corte da CA para identificar crianças com risco de obesidade.

**Métodos:**

Um estudo multicêntrico, prospectivo, tranversal foi realizado em crianças com idades entre 6 e 10 anos, matriculadas no ensino fundamental de escolas públicas e particulares de 13 cidades do estado de São Paulo. A estatura, o peso e a CA foram medidos em duplicata em 22.000 crianças (11.199 meninos). Para estabelecer o melhor ponto de corte da CA para o diagnóstico da obesidade, foram calculadas curvas ROC com crianças classificadas como com peso normal e obesas, de acordo com as curvas do IMC, estratificadas por gênero e idade, e o índice Youden foi utilizado como a eficácia potencial máxima desse biomarcador. Valores de p < 0,05 foram considerados estatisticamente significativos.

**Resultados:**

Os valores da CA aumentaram com a idade, tanto nos meninos quanto nas meninas. A prevalência de obesidade em cada grupo variou de 17% (6 anos de idade) a 21,6 % (9 anos de idade), dentre os meninos, e de 14,1% (7 anos de idade) a 17,3 % (9 anos de idade), dentre as meninas. As análises ROC mostraram o percentil 75 como ponto de corte para o risco de obesidade, e o diagnóstico de obesidade está classificado no percentil 85 ou acima.

**Conclusão:**

Curvas de referência da CA específicas para idade e sexo em crianças brasileiras e pontos de corte para o risco de obesidade podem ser usados em triagem nacional e estudos intervencionais para reduzir a carga da obesidade no Brasil. (Arq Bras Cardiol. 2020; 114(3):530-537)

## Introdução

A prevalência da obesidade ao redor do mundo, particularmente dentre as crianças, vem aumentando exponencialmente. Durante os últimos 30 anos, várias pesquisas nacionais registraram aumento significativo na prevalência de obesidade e sobrepeso em todas as regiões brasileiras.^1^ A obesidade infantil está relacionada ao desenvolvimento da obesidade nos adultos. Além disso, a criança obesa está exposta a maiores riscos de desenvolver o diabetes tipo 2, hipertensão e anormalidades vasculares, que são considerados precursores da aterosclerose na idade adulta. A hipertrofia do ventrículo esquerdo e os problemas renais também têm sido descritos nas crianças obesas.^2^ O aumento da prevalência da obesidade e a forte associação com diversas comorbidades em crianças demonstram a relevância para a saúde pública.^3^ Consequentemente, é necessário encontrar um parâmetro antropométrico simples que possa ser utilizado para identificar crianças obesas ou com risco de se tornarem obesas, que possa contribuir para o desenvolvimento de ferramentas apropriadas de intervenção voltadas para a melhoria dessa tendência.

As taxas de obesidade e sobrepeso nos grupos populacionais são tipicamente fundamentadas no índice de massa corporal (IMC). Entretanto, o IMC não reflete a composição corporal, oferendo informações limitadas sobre a adiposidade central ou abdominal. A medição da circunferência abdominal (CA) tem alta sensibilidade e é bastante eficaz na predição de níveis de adiposidade visceral na população pediátrica. De fato, os valores da CA se correlacionam com os distúrbios metabólicos relacionados à obesidade, incluindo a resistência à insulina, a dislipidemia e a hipertensão. Sugeriu-se que a medição da CA poderia substituir o IMC na avaliação de risco para comorbidades relacionadas com a obesidade nas populações jovens.^4-7^

Já que os valores da CA são influenciados pela idade, sexo, diferenças étnicas e geopolíticas, estudos disponíveis apresentaram a distribuição de percentis da CA em crianças e adolescentes de um grande número de países.^8-29^ Tomando isso como base, pretendemos reforçar o conjunto de informações já relatado em alguns estudos nacionais sobre esse assunto,^30-37^ por meio da descrição da distribuição de percentis da CA e do cálculo de pontos de corte da CA capazes de prever a obesidade em uma ampla amostra de crianças, de 6 a 10 anos de idade, no estado de São Paulo. Além disso, comparamos os percentis atuais da CA com dados obtidos a partir de estudos realizados em outros países e de outro estudo brasileiro, que avaliaram população semelhante, em função da idade e do sexo.

## Métodos

### Desenho e população do estudo

Este é um estudo longitudinal, prospectivo, multicêntrico,, iniciado em março de 2010 e finalizado em julho de 2010. O estudo teve apoio científico da Sociedade de Cardiologia do Estado de São Paulo (SOCESP), e foi coordenado regionalmente pela SOCESP - unidade regional de Araras, SP.

O presente estudo teve como objetivo incluir pelo menos 30% de crianças de ambos os sexos, com idades entre 6 e 10 anos, matriculadas em escolas públicas e privadas do ensino fundamental de 13 cidades do município de Araras, localizado no estado de São Paulo, Brasil. De acordo com dados da Sinopse Estatística da Educação, fornecidos pelo Instituto Nacional de Estudos e Pesquisas Educacionais Anísio Teixeira (INEP), o número de crianças matriculadas em todas as cidades incluídas no presente estudo era de 63.891, em 2010. Para excluir as crianças que não frequentavam a escola e alunos com necessidades especiais acima de 10 anos de idade (que ultrapassariam a idade limite deste estudo), utilizamos um fator de correção de 10% da amostra total. Assim, a população total estimada de crianças de 6 a 10 anos de idade foi de 57.501 nessas localidades.

As Secretarias de Educação e as escolas municipais e privadas de todos os municípios foram contactadas. O estudo foi realizado em locais nos quais houve aprovação em todas as instâncias: 147 escolas públicas e 14 escolas privadas. A permissão da coordenação das escolas e dos pais para participarem da avaliação foi devidamente assegurada e cada centro participante teve que seguir as diretrizes éticas e de gerenciamento de dados da instituição.

A idade das crianças foi foi registrada no estudo de Rousham et al.,^38^ Consequentemente, 15 de junho do ano de nascimento foi considerada a data estimada de nascimento.

### Comparação entre as medições antropométricas e percentis da CA

Pesquisadores treinados realizaram as medições de acordo com procedimentos padronizados.

As medições antropométricas foram efetuadas com as crianças vestindo roupas leves e sem sapatos. A estatura e o peso foram mensurados em duplicata por meio de uma balança eletrônica digital, equipada com um estadiômetro portátil, com precisão de 0,1 cm e 0,1 kg, respectivamente. A média das duas medições foi usada para calcular o IMC [IMC = peso (kg)/altura(cm)^2^]. Os meninos e as meninas foram classificados segundo as faixas de percentis do IMC (as curvas do IMC estabelecidas para cada sexo e idade), utilizando os parâmetros das curvas para a população definidas pelo NCHS-CDC (Centro Nacional de Estatística para a Saúde - Centro para Controle e Prevenção de Doenças - EUA).^39^

O estado nutricional das crianças foi categorizado de acordo com os percentis do IMC: obesos (IMC > 95%), com sobrepeso (IMC entre 85 e 95) e peso normal (IMC < 85%).

A circunferência abdominal foi aferida em duplicata, na metade da distância entre a última costela e a crista ilíaca superior, com uma fita métrica não-flexível, em posição vertical, com o abdomen relaxado ao final de uma expiração suave.^40^

Para comparar a atual distribuição de percentis da CA, realizamos uma revisão da literatura de estudos de base populacional que haviam avaliado esse parâmetro em um grupo etário semelhante. Para comparações entre os países, utilizamos o percentil 50 da CA em função da idade. Nossos dados também foram comparados com um estudo brasileiro anterior, para identificar tendências nos valores da cinrcunferência abdominal ao longo do tempo.

### Análise estatística

Os dados antropométricos da população pediátrica são apresentados como média com desvio padrão (DP), mediana, percentis e valores mínimos e máximos. A correlação linear (coeficiente de correlação de Pearson) ou a correlação não-linear (coeficiente logarítmico, inverso, quadrático, cúbico, composto, de potência, sigmóide, de crescimento e exponencial) foi calculada por regressão. Quando relevante, o coeficiente linear ou não-linear mais alto foi utilizado para determinar o melhor modelo que explicasse o fenômeno. Para estabelecer o melhor ponto de corte da CA para o diagnóstico da obesidade, geramos uma curva ROC com crianças com peso normal e obesas, utilizando os percentis do IMC, estratificadas por sexo e idade, e utilizamos a maior soma de sensibilidade e especificidade para estabelecer o ponto de corte (Índice de Youden).^41^ Valores de p < 0,05 foram considerados relevantes. Os dados foram analisados usando o software IBM SPSS Statistics versão 23.0 (IBM Inc., Armonk, NY, EUA).

## Resultados

Ao todo, 22.000 crianças (11.199 meninos e 10.886 meninas) foram incluídas, o que representa mais de 30% da população estimada, variando de 1.606 a 2.610 meninos e 1.612 a 2.502 meninas, para cada um dos dos cinco períodos de idade de 6 a 10 anos. As características antropométricas basais médias separadas por idade e sexo estão apresentadas na Tabela 1. Observou-se esperado aumento progressivo no peso, estatura, IMC e CA em ambos os sexos de 6 a 10 anos. A prevalência de obesidade em cada faixa etária variou entre um mínimo de 17% (6 anos de idade) e um máximo de 21,6% (9 anos de idade), dentre os meninos, e um mínimo de 14,1% (7 anos de idade) e um máximo de 17,3% (9 anos de idade), dentre as meninas (Tabela 1). Aproximadamente 30% dos meninos e meninas apresentaram excesso de gordura, e foram classificados como com sobrepeso ou obesos.

**Tabela 1:** – Valores médios antropométricos, desvio padrão e prevalência de sobrepeso e obesidade em função da idade e do sexo

Meninos Idade (y)	n	Peso (Kg)	Altura (cm)	IMC (kg/m^2^)	CA (cm)	Prevalência de sobrepeso (%)	Prevalência de obesidade (%)
6	1,606	24,.5 ± 5,85	1,20 ± 0,06	16,7 ± 2,83	58,8 ± 7,63	11,8	17
7	2,223	26,8 ± 6,76	1,25 ± 0,07	16,9 ± 3,14	60,5 ± 8,37	11,6	18,7
8	2,450	29,5 ± 7,81	1,30 ± 0,07	17,3 ± 3,41	62,1 ± 8,80	12,3	18,8
9	2,610	33,1 ± 9,06	1,35 ± 0,07	17,9 ± 3,70	64,4 ± 10,15	13,0	21,6
10	2,310	36,8 ± 10,37	1,40 ± 0,08	18,4 ± 3,92	67,2 ± 10,54	14,0	20,4
Total	11,199						
**Meninas Idade (y)**							
6	1,612	24,2 ± 5,85	1,19 ± 0,06	16,7 ± 2,95	59 ± 7,95	13,6	15,0
7	2,236	26,0 ± 6,80	1,23 ± 0,06	16,8 ± 3,15	59,8 ± 8,43	12,2	14,1
8	2,284	29,2 ± 7,85	1,29 ± 0,07	17,2 ± 3,49	61,9 ± 9,16	13,5	16,6
9	2,502	32,8 ± 8,92	1,35 ± 0,07	17,8 ± 3,54	64,1 ± 9,75	15,6	17,3
10	2,252	36,9 ± 9,96	1,41 ± 0,08	18,3 ± 3,76	66,8 ± 10,19	14,8	15,7
Total	10,886						

y: nos de idade

**Figura 1:**
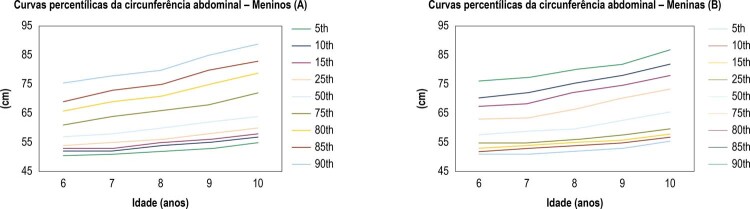
– *Curvas percentílicas da CA de crianças brasileiras (6–10 anos de idade). Meninos (A); meninas (B).*

A Figura 1 mostra as curvas percentílicas suavizadas da CA (5, 10, 25, 50, 75, 85, 90 e 95) para meninos e meninas.

Analisamos a correlação entre os parâmetros da CA e antropométricos. Houve uma forte correlação entre a CA e o peso (r^2^ = 0,77, p < 0,001) e a CA e o IMC (r^2^ = 0,74, p < 0,001) e uma correlação fraca entre a CA e a estatura (r^2^ = 0,31, p < 0,001).

A distribuição dos valores da CA em percentis de 5 a 95, em função da idade e do sexo, bem como os pontos de corte da CA com mais alta sensibilidade e especificidade, identificados pela curva ROC, podem ser observados na Tabela 2.

**Tabela 2:** – Distribuição dos pencentis e pontos de corte da CA para obesidade em função da idade e do sexo da população estudada

	Pontos de corte (cm) para obesidade	Percentis da CA								
**Meninos Idade (y)**		**5**	**10**	**15**	**25**	**50**	**75**	**85**	**90**	**95**
6	61,2	50,5	52,0	53,0	54,0	57,0	61,0	66,0	69,0	75,8
7	63,2	51,0	52,0	53,0	55,0	58,0	64,0	69,0	73,0	78,0
8	64,8	52,0	54,0	55,0	56,0	60,0	66,0	71,0	75,0	80,0
9	67,7	53,0	55,0	56,0	58,0	62,0	68,0	75,0	80,0	85,0
10	70,5	55,0	57,0	58,0	60,0	64,0	72,0	79,0	83,0	89,0
**Meninas Idade (y)**										
6	62,7	50,0	51,0	52,0	54,0	57,0	62,5	67,0	70,0	76,0
7	64,2	50,0	52,0	53,0	54,0	58,0	63,0	68,0	71,7	77,0
8	64,7	51,0	53,0	54,0	55,2	59,0	66,0	72,0	75,0	80,0
9	69,7	52,0	54,0	55,0	57,0	62,0	70,0	74,5	78,0	82,0
10	72,7	54,5	56,0	57,0	59,0	64,8	73,0	78,0	82,0	87,0

y: anos de idade.

Os pontos de corte da CA ficaram ligeiramente abaixo ou na faixa de 75. Assim sendo, para as crianças que tiveram a CA classificada no percentil 75, a presença de sobrepeso ou obesidade deve ser considerada. Além disso, o diagnóstico de obesidade está claramente presente nas crianças com a CA classificada no percentil 85 ou acima.

Neste estudo, dentre as crianças eutróficas, menos de 7% apresentaram valor da CA que indicasse obesidade. Dentre as crianças categorizadas como obesas pelo IMC, quase 90% podem ser caracterizadas como sendo obesas, simplesmente através da mensuração da CA (Tabela 3).

**Tabela 3:** – Aplicação dos valores de corte da cintura para obesidade na população do estudo classificada pelo IMC como não obesa e obesa, em função da idade e do sexo

	Crianças não obesas (%)		Crianças obesas (%)	
**Meninos Idade (y)**	≤ Ponto de corte *	> Ponto de corte *	≤ Ponto de corte *	> Ponto de corte *
6	92,9	7.1	11,0	89,0
7	95,7	4.2	8,4	91,6
8	94,1	5.9	9,1	90,9
9	96,7	3.3	12,6	87,4
10	94,2	5.8	10,6	89,4
**Meninas Idade (y)**				
6	93,1	6,9	9,9	90,1
7	95,4	4,6	4,7	95,3
8	91,5	8,5	4,9	95,1
9	95,8	4,2	11,8	88,2
10	94,6	5,4	12,1	87,9

y: anos de idade; *: valor de ponto de corte da CA pré-determinado para cada idade ou sexo.

**Figura 2:**
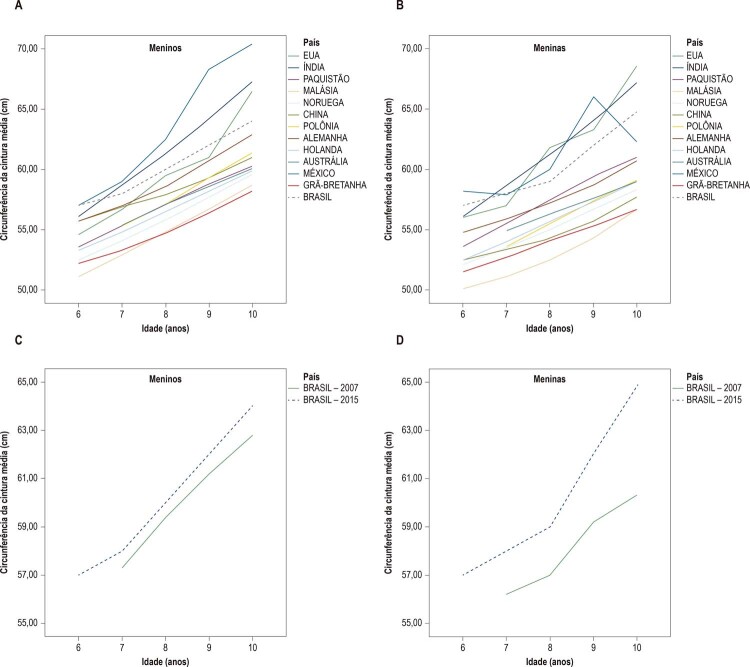
– *Comparação entre as curvas da CA (percentil 50) nas crianças de 13 países diferentes. A: Comparação entre meninos de 6 a 10 anos; B: Comparação entre meninas de 6 a 10 anos. C: Comparação entre as curvas da CA (percentil 50) de meninos (C) e meninas (D) das diferentes regiões brasileiras estudadas.*

A Figura 2 mostra a representação gráfica dos valores do percentil 50 (cm) da CA estabelecidos no presente estudo juntamente com os valores obtidos de publicações de 12 países diferentes, para meninos (A) e meninas (B), com idades entre 6 e 10 anos. Detectamos que os meninos brasileiros de 6 anos de idade tiveram uma CA semelhante aos meninos mexicanos, a mais alta dentre todos os países. Os meninos brasileiros de 7 a 9 anos de idade apresentaram valores de CA menores do que aqueles obsevados nos meninos mexicanos e indianos e, aos 10 anos de idade, os valores também foram menores em relação aos meninos dos EUA. As meninas brasileiras de 6, 7 e 10 anos de idade tiveram valores semelhantes ou ligeiramente menores de CA do que aqueles detectados nas meninas mexicanas e indianas, e as meninas de 8 e 9 anos de idade também tiveram valores da CA menores quando comparadas com as meninas mexicanas.

Adicionalmente, a Figura 2 apresenta o percentil 50% dos valores da CA de meninos (C) e meninas (D) brasileiros em um estudo anterior, publicado em 2007. Pode-se observar que as curvas percentílicas atuais (50) nos meninos brasileiros foram mais altas na comparação com os valores de 2007. As curvas percentílicas atuais (50) nas meninas brasileiras são muito mais elevadas do que os valores obtidos em 2007, em torno de 2,0 cm, aos 7 e 8 anos; 2,5 cm, aos 9 anos de idade, chegando a 4,0 cm aos 10 anos.

## Discussão

Este estudo apresenta os valores percentílicos específicos da CA, em função da idade e do sexo, em uma amostra ampla e representativa de crianças brasileiras, com idades entre 6 e 10 anos, com base em uma avaliação antropométrica multicêntrica e longitudinal de crianças em idade escolar. Além disso, é o primeiro estudo a propor que os pontos de corte da CA estão associados à obesidade, segundo o IMC de meninos e meninas de 6 a 10 anos de idade. Ademais, nosso estudo demonstrou que, nessa população escolar, a prevalência de excesso de gordura ficou em torno de 30%, com 15% dos meninos e meninas com sobrepeso e outros 15% já considerados obesos. De fato, esses resultados corroboram dados anteriores que indicaram que a obesidade infantil é um problema de saúde cada vez mais sério nacional e mundialmente.^42-44^

Esses dados complementam o conjunto já existente de valores de referência da CA obtidos em alguns outros países e aprimoram as capacidades de avaliação da obesidade infantil, nos mais diversos locais para a assistência à criança. Já que várias ligações podem ser estabelecidas com os valores da circunferência abdominal, tais como depósito de gordura intra-abdominal e fatores de risco cardiovascular nas crianças,^45-48^ a circunferência abdominal poderia ser adotada como uma medida alternativa ou complementar ao IMC nas crianças. A forte correlação encontrada entre os valores da circunferência e o IMC neste estudo demonstra que tal substituição ou uso adicional é viável.

Além disso, os pontos de corte da CA propostos para a obesidade indicam uma forte aproximação, tanto do peso normal (eutrófico) quanto da obesidade, conforme definidos pela categorização internacional do IMC. Menos de 7% das crianças eutróficas teriam um um ponto de corte da CA mais alto, o que indicaria um IMC elevado. Nesses casos, outras explicações potencias podem estar presentes, tais como excesso de gordura inicial caracterizado por obesidade central ou alterações clínicas adicionais (tais como distúrbios gastrointestinais, dentre outros). Em relação às crianças obesas, quase 87% poderiam ter sido diagnosticadas com obesidade apenas por meio da mensuração da CA. Além disso, a CA é uma ferramenta simples e viável de mensuração, que requer equipamentos de baixo custo e acessíveis, e pouco conhecimento técnico, tornando possível realizar mensurações em períodos programados em todas as escolas. Nossos resultados demonstram que as crianças brasileiras apresentam valores elevados de percentil 50 para a CA, com os resultados dos meninos ocupando aproximadamente o terceiro lugar e, das meninas, o quarto lugar dentre todos os países analisados. Mais importante ainda, entrelaçando os valores de 50% do percentil da CA obtidos em 2007 com os valores atuais, confirmamos o incremento dos valores em todas as idades nos meninos e, com mais intensidade, nas meninas.

Enquanto a Federação Internacional de Diabetes (IDF) sugere que a síndrome metabólica (MetS) não deveria ser diagnosticada em crianças menores de 10 anos, a perda de peso deveria ser considerada para aquelas crianças com obesidade abdominal, conforme medição da circunferência abdominal. A correlação do tecido adiposo visceral e a circunferência abdominal nas crianças foi confirmada e é um preditor importante de resistência à insulina, níveis lipídicos e pressão arterial – todos componentes da MetS.

A definição consensual da IDF de MetS em crianças e adolescentes teve como objetivo definir uma caracterização aceita universalmente para facilitar o diagnóstico da MetS e acelerar as medidas preventivas, antes que a criança ou o adolescente desenvolva diabetes ou doença cardiovascular. A obesidade, especialmente na região abdominal, está associada ao aumento do risco de doença cardiovascular e diabetes tipo 2.^49^

O presente estudo delimitou uma área de 12 cidades do estado de São Paulo. Acreditamos que ele possa ser representativo das crianças em idade escolar de uma grande área do Brasil, uma vez que incluiu o número mais significativo de crianças de escolas particulares e públicas já relatado no Brasil. Além disso, o estado de São Paulo tem um alto grau de miscigenação, e seu interior tem um desenvolvimento socioeconômico comparável com as regiões sul e sudeste e até com muitas áreas mais próximas do centro-oeste. Como enfatizado em vários relatos, a avaliação dos distúrbios cardiometabólicos nas crianças só é viável mediante a disponibilidade de referências específicas à associação entre idade, sexo e origem étnica e riscos para a saúde. Com uma amostra de 9.713 indivíduos de 2 a 18 anos de idade, incluindo 3.414 afro-americanos, 2.746 euro-americanos, e 3.553 méxico-americanos (MA), Fernandez et al.^14^descreveram e forneceram estimativas de distribuição de curvas percentílicas da CA amplamente utilizadas em diferentes países, o conhecido banco de dados do NHANES. Nos percentis 75 e 90, as meninas MA apresentam o aumento total mais rápido dentre todas as meninas. Em todos os percentis considerados, os indivíduos MA apresentaram a CA total mais elevada e o aumento total da taxa da CA mais rápido com a idade. Esse dado sustenta os percentis da CA elevados dentre as crianças brasileiras e o aumento mais significativo dentre as meninas brasileiras.

Com base em dados robustos coletados, Fernandez et al.^14^enfatizaram que atenção especial deveria ser concentrada nas crianças cujos valores da CA encontram-se no percentil 75 e 90, uma vez que essa redução ajuda a identificar crianças com risco de várias comorbidades e sugerem fortemente ações de prevenção [contra essas situações]. Todos os valores percentílicos encontrados no nosso estudo foram maiores comparados com aqueles descritos por Fernandez et al.,^14^ Dentre as crianças alemães (6-10-meninos e meninas, entre 6 e 10 anos de idade), o percentil 97 da CA estava associado à obesidade abdominal.^50^

Hábitos de estilo de vida saudável, como adesão a uma dieta saudável e prática de atividade física, podem baixar o risco de obesidade e de doenças relacionadas.^51,52^ Os hábitos alimentares e de atividade física de crianças e adolescentes são influenciados por muitos fatores sociais, incluindo a família, a comunidade, a escola, ambientes de cuidado infantil, prestadores de cuidado de saúde, instituições religiosas, agências governamentais, a mídia, as indústrias de bebidas, alimentos e entretenimento.^53-56^ As escolas desempenham uma função particularmente crítica para o estabelecimento de um ambiente seguro e acolhedor, com políticas e práticas que incentivem os comportamentos saudáveis. Elas também oferecem oportunidades para os alunos aprenderem e praticarem hábitos alimentares saudáveis e atividade física.^56^ A importância do presente estudo foi fornecer valores representativos da CA das crianças brasileiras que possam ser utilizados como uma ferramenta de avaliação que auxilie no cumprimento das recomendações da saúde pública.

Outra contribuição relevante para o desenvolvimento de dados epidemiológicos nacionais é o Estudo de Riscos Cardiovasculares em Adolescentes (ERICA).^57^ Trata-se de um grande estudo transversal, nos níveis nacional e escolar, que tem como objetivo avaliar a prevalência dos fatores de risco cardiovasculares, incluindo os componentes da síndrome metabólica, em aproximadamente 85.000 alunos, com idades entre 12 e 17 anos. Uma publicação recente do ERICA descreveu a prevalência da síndrome metabólica como algo em torno de 2,6%, e as seguintes combinações de elementos mais comuns, correspondendo a 3/4 das combinações: circunferência abdominal aumentada (CA), níveis baixos de colesterol HDL e pressão arterial alta; seguidas de CA aumentada, níveis baixos de colesterol HDL e níveis elevados de triglicerídeo; e CA aumentada, níveis baixos de colesterol HDL, níveis elevados de triglicerídeo, e pressão arterial alta. Consequentemente, os resultados do ERICA reforçam a importância da CA como um potencial indicador de um desordenamento mais global que poderia determinar a síndrome metabólica.

A principal limitação deste estudo é a descrição das curvas percentílicas com base em uma amostra de crianças de apenas um estado, São Paulo, e não derivadas de regiões de todo o Brasil selecionadas aleatoriamente. Esse fato pode restringir a generalização dos resultados para crianças de todo o Brasil. Além disso, para validar os pontos de corte de sobrepeso e obesidade, é necessário testar os respectivos valores em uma coorte diferente de crianças.

## Conclusão

As curvas de referência para a CA específicas em função da idade e do sexo nas crianças brasileiras e os pontos de corte para risco de obesidade podem ser utilizados para triagem nacional e estudos intervencionais com vistas a reduzir a carga de obesidade no Brasil.
