# Supplementary material for: Os Percentis e Pontos de Corte da Circunferência Abdominal para Obesidade em uma Ampla Amostra de Estudantes de 6 a 10 Anos de Idade do Estado de São Paulo, Brasil
Source: Arq Bras Cardiol. 2020 Apr 6;114(3):530–7. [Article in Portuguese] doi: 10.36660/abc.20190043 (PMC7792724; doi:10.36660/abc.20190043)
Supplement: Translation

# Translation: Waist Circumference Percentiles and Cut-Off Values for Obesity in a Large Sample of Students from 6 To 10 Years Old Of The São Paulo State, Brazil

**Authors:** José Luiz F Santos, Valentin P. Valério, Rafael N. Fernandes, Ligia Duarte, Antonio C. Assumpção, Jayme Guerreiro, Antonio L. Sickler, Álvaro A. R. Lemos, Jayro G Goulart, Luiz Antonio Machado Cesar, Ibraim Masciarelli Pinto, Carlos Magalhães, Maria Fernanda Hussid, Cleber Camacho, Alvaro Avezum, Carine T. Sangaleti, Fernanda Marciano Consolim-Colombo

**Affiliations:** 1 BrazilSociedade de Cardiologia do Estado de São Paulo, São Paulo, SP – Brazil; 2 BrazilInstituto do Coração (InCor) – Cardiopneumologia, São Paulo, SP – Brazil; 3 BrazilInstituto Dante Pazzanese de Cardiologia – Cardiologia, São Paulo, SP – Brazil; 4 BrazilUniversidade Nove de Julho, São Paulo, SP – Brazil; 5 BrazilUniversidade Estadual do Centro-Oeste - Enfermagem, Guarapuava, PR – Brazil; 6 BrazilUniversidade de Sao Paulo Faculdade de Medicina Hospital das Clínicas Instituto do Coração, São Paulo, SP – Brazil

**Keywords:** Child, Waist Circunference/physiology, Obesity, Students, Parameters, Anthropometry

## Abstract

**Backgroud:**

The prevalence of obesity has systematically been increased in the population, including children and adolescents, around the world.

**Objectives:**

To describe reference percentile curves for waist circumference (WC) in Brazilian children and provide cut-off values of WC to identify children at risk for obesity.

**Methods:**

A multicenter, prospective, cross-sectional study was performed with children aged from 6 to 10 years old, enrolled in public and private elementary schools from 13 cities of the São Paulo State. Height, weight, and WC were measured in duplicate in 22,000 children (11,199 boys). To establish the WC best cut-off value for obesity diagnosis, ROC curves with children classified as normal weight and obese were calculated, according to BMI curves, stratified by gender and age, and the Youden Index was utilized as the maximum potential effectiveness of this biomarker. A p < 0.05 was considered statistically significant.

**Results:**

WC values increased with age in both boys and girls. The prevalence of obesity in each age group varied from 17% (6 years old) to 21.6% (9 years old) among boys, and from 14.1% (7 years old) to 17.3 % (9 years old) among girls. ROC analyses have shown the 75^th^ percentile as a cut-off for obesity risk, and the diagnosis of obesity is classified on the 85^th^ percentile or more.

**Conclusion:**

Age and gender specific reference curves of WC for Brazilian children and cut-off values for obesity risk may be used for national screening and interventional studies to reduce the obesity burden in Brazil. (Arq Bras Cardiol. 2020; 114(3):530-537)

## Introduction

The worldwide prevalence of obesity, particularly among children, has been increasing exponentially. Over the last 30 years, several national surveys have recorded significant increment in the prevalence of obesity and overweight across Brazilian regions.^1^ Childhood obesity is linked to the development of obesity in adults. Moreover, the obese child is exposed to a higher risk of developing type 2 diabetes, hypertension and vascular abnormalities, which are considered precursors of atherosclerosis in adulthood. Left ventricular hypertrophy and kidney problems have also been described in obese children.^2^ The increased prevalence of obesity and the strong association with several comorbidities in children provide the relevance for public health.^3^ As a result, it is necessary to find a simple anthropometric parameter that may be used to identify obese or at- risk- of- becoming obese children, which may contribute to the appropriate intervention tools to improve this trend.

Obesity and overweight rates across population groups are typically based on body mass index (BMI). However, BMI does not reflect body composition, providing limited information on the central or abdominal adiposity. Waist circumference (WC) measurement is highly sensitive and quite effective in predicting visceral adiposity levels in the pediatric population. Indeed, WC values correlate with obesity-related metabolic disorders, including insulin resistance, dyslipidemia and hypertension. It has been suggested that WC measurement could replace BMI in risk assessment for obesity-related comorbidities among young populations.^4-7^

Since WC values are influenced by age, gender, ethnic and geopolitical differences, available studies have presented the distribution of WC percentiles of children and adolescents of a wide range of countries.^8-29^ Based on the above-mentioned, we intended to boost the set of information already reported by some national studies on this matter^30-37^by describing the WC percentile distribution and by calculating the WC cut-off values which would be able to predict obesity in a large sample of children aged from 6 to 10 years in the São Paulo State. Moreover, we compared the current WC percentile with data obtained from studies performed in other countries and another Brazilian study evaluating a similar population, according to age and gender.

## Methods

### Design and study population

This is a longitudinal, prospective, multicenter study, initiated in March 2010 and concluded in July 2010. The study was scientifically supported by the São Paulo Society of Cardiology (Sociedade de Cardiologia do Estado de São Paulo, SOCESP), and was regionally coordinated by the SOCESP Regional Directory Office of Araras, SP.

The current study aimed to include at least 30% of children of both genders, aged from 6 to 10 years old, enrolled at public and private elementary schools of the 13 cities of the Araras District of São Paulo State, Brazil. According to the Statistical and Educational Nationwide Survey Data provided by the National Institute of Educational Studies Anisio Teixeira (INEP), the number of children enrolled in all the cities included in the present study was 63,891 in 2010. In order to exclude children who did not attend school and special needs students older than 10 years of age (which would go beyond the age limit of this study) we used a correction factor of 10% of the total sample. Thus, the total estimated population of children from 6 to 10 years old was 57,501 in these localities.

The Secretariats of Education and the municipal and private schools of all Municipalities were contacted. The study was carried out in places where there was an approval in all instances: 147 public schools and 14 private schools. The Schools' coordination and parent’s permission to participate in the evaluation was duly secured and each participating center had to comply with the ethical and data management guidelines of the local institution.

The children´s age was reported by Rousham et al.^38^ study. Therefore, 15 June of the year birth was taken as an estimated date of birth.

### Anthropometry measurements and WC percentiles comparison

Trained researchers performed the measurements according to standardized procedures.

Anthropometric measurements were undertaken with the children wearing light clothing and no shoes. The height and weight was measured in duplicate with a digital electronic scale provided with a portable stadiometer, to the nearest 0.1 cm and 0.1 kg, respectively. The average of the two measurements was used to calculate the BMI [BMI = weight (kg)/height(cm)^2^]. Boys and girls were classified according to the BMI percentile ranges (the BMI curves established for each gender and age), using the parameters of the population curves of NCHS-CDC (National Center for Health Statistics - Centers for Disease Control and Prevention - USA).^39^

The nutritional state of the children was categorized according to the BMI percentiles: obese (BMI > 95%), overweight (BMI between 85 and 95%) and normal weight (BMI < 85%).

Abdominal circumference was measured in duplicate, at half the distance between the lowest rib and the superior border of the iliac crest, with a non-flexible tape, in the upright position, with the abdomen relaxed at the end of gentle expiration.^40^

To compare the actual WC percentiles distribution, we performed a literature review of the population-based studies that had evaluated this parameter in a similar age group. For comparisons among countries, we used the 50^th^ percentiles of WC according to age. Our data has also been compared with a previous Brazilian study, to identify trends in abdominal circumference values in time.

### Statistical analysis

Anthropometrical data from the pediatric population are presented as mean ± standard deviation (SD), median, percentiles and minimum and maximum values. The linear (Pearson’s correlation coefficient) or non-linear correlation (logarithmic, inverse, quadratic, cubic, compound, power, sigmoid, growth and exponential coefficient) was calculated by regression. If significant, the higher linear or non-linear coefficient was used to identify the best model which explains the phenomenon. To establish the WC best cut-off value for obesity diagnosis, we generated a ROC Curve with normal weight and obese children using the BMI percentiles, stratified by gender and age, and used the highest sum of sensitivity and specificity to set the cut-off point (Youden Index).^41^ A p < 0.05 was considered significant. The data were analyzed using IBM SPSS Statistics for Windows, Version 23.0 from IBM Corp. Released in 2015. Armonk, NY, USA.

## Results

A total of 22,000 children (11,199 boys and 10,886 girls) were included, which represents more than 30% of the estimated population, ranging from 1,606 to 2,610 boys and from 1,612 to 2,502 girls for each of the five periods of age, from 6 to 10 years old. The mean baseline anthropometric characteristics separated by age and gender are presented in Table 1. There was an expected progressive increase in weight, height, BMI and WC in both genders from 6 to 10 years. The prevalence of obesity in each age group varied from a minimum of 17 % (6 years old) to a maximum of 21.6% (9 years old) among boys, and a minimum of 14.1% (7 years old) to a maximum of 17.3 % (9 years old) among girls (Table 1). Approximately 30% of boys and girls had excess of fat, and were classified as either overweight or obese.

**Table 1:** – Anthropometric mean values, standard deviation and prevalence of overweight and obesity according to age and gender

Boys Age (y)	n	Weight (Kg)	Height (cm)	BMI (kg/m2)	WC (cm)	Overweight prevalence (%)	Obesity prevalence (%)
6	1.606	24.5 ± 5.85	1.20 ± 0.06	16.7 ± 2.83	58.8 ± 7.63	11.8	17
7	2.223	26.8 ± 6.76	1.25 ± 0.07	16.9 ± 3.14	60.5 ± 8.37	11.6	18.7
8	2.450	29.5 ± 7.81	1.30 ± 0.07	17.3 ± 3.41	62.1 ± 8.80	12.3	18.8
9	2.610	33.1 ± 9.06	1.35 ± 0.07	17.9 ± 3.70	64.4 ± 10.15	13.0	21.6
10	2.310	36.8 ± 10.37	1.40 ± 0.08	18.4 ± 3.92	67.2 ± 10.54	14.0	20.4
Total	11.199						
**Girls Age (y)**							
6	1.612	24.2 ± 5.85	1.19 ± 0.06	16.7 ± 2.95	59 ± 7.95	13.6	15.0
7	2.236	26.0 ± 6.80	1.23 ± 0.06	16.8 ± 3.15	59.8 ± 8.43	12.2	14.1
8	2.284	29.2 ± 7.85	1.29 ± 0.07	17.2 ± 3.49	61.9 ± 9.16	13.5	16.6
9	2.502	32.8 ± 8.92	1.35 ± 0.07	17.8 ± 3.54	64.1 ± 9.75	15.6	17.3
10	2.252	36.9 ± 9.96	1.41 ± 0.08	18.3 ± 3.76	66.8 ± 10.19	14.8	15.7
Total	10.886						

y: years old.

Figure 1 shows the smoothed computed waist circumference percentile curves for the 5^th^, 10^th^, 25^th^, 50^th^, 75^th^, 85^th^, 90^th^ and 95^th^ percentile for boys and girls.

**Figure 1:** – WC percentile curves for Brazilian children (aged 6–10 years). Boys (A) Girls (B).

We analyzed the correlation between WC and anthropometric parameters. There was a strong correlation between WC and weight (r^2^ = 0.77, p < 0.001) and WC and BMI (r^2^ = 0.74, p < 0.001), and a weak correlation between WC and height (r^2^ = 0.31, p < 0.001).

The distribution of WC values in percentiles from 5^th^ to 95^th^, according to age and gender, as well as the best sensitivity and specificity ROC cut-off values of WC, are shown in Table 2.

**Table 2:** – Distribution of WC percentiles and cut-off points for obesity according to age and gender in the population studied

	Cut-off values (cm) for obesity	WC percentiles								
**Boys Age (y)**		**5th**	**10th**	**15th**	**25th**	**50th**	**75th**	**85th**	**90th**	**95th**
6	61.2	50.5	52.0	53.0	54.0	57.0	61.0	66.0	69.0	75.8
7	63.2	51.0	52.0	53.0	55.0	58.0	64.0	69.0	73.0	78.0
8	64.8	52.0	54.0	55.0	56.0	60.0	66.0	71.0	75.0	80.0
9	67.7	53.0	55.0	56.0	58.0	62.0	68.0	75.0	80.0	85.0
10	70.5	55.0	57.0	58.0	60.0	64.0	72.0	79.0	83.0	89.0
**Girls Age (y)**										
6	62.7	50.0	51.0	52.0	54.0	57.0	62.5	67.0	70.0	76.0
7	64.2	50.0	52.0	53.0	54.0	58.0	63.0	68.0	71.7	77.0
8	64.7	51.0	53.0	54.0	55.2	59.0	66.0	72.0	75.0	80.0
9	69.7	52.0	54.0	55.0	57.0	62.0	70.0	74.5	78.0	82.0
10	72.7	54.5	56.0	57.0	59.0	64.8	73.0	78.0	82.0	87.0

y: years old.

The cut-off values for WC are slightly below or in the range of 75^th^. Thus, for children with a WC classified into the 75^th^ percentile the presence of overweigth or obesity must be taken into account. Moreover, the diagnosis of obesity is cleary present in children with a WC classified in the 85^th^percentile or higher.

In this study, among the eutrophic children, fewer than 7% had a WC value indicating obesity. Among the children categorized as obese by BMI, almost 90% may be characterized as being obese simply by measuring the WC (Table 3).

**Table 3:** – Application of the obesity waist cut-off values in the study population classified by BMI as non-obese and obese, according to age and gender

	Non-obese children (%)		Obese children (%)	
**Boys Age (y)**	≤ Cut-off *	> Cut-off *	≤ Cut-off *	> Cut-off *
6	92.9	7.1	11.0	89.0
7	95.7	4.2	8.4	91.6
8	94.1	5.9	9.1	90.9
9	96.7	3.3	12.6	87.4
10	94.2	5.8	10.6	89.4
**Girls Age (y)**				
6	93.1	6.9	9.9	90.1
7	95.4	4.6	4.7	95.3
8	91.5	8.5	4.9	95.1
9	95.8	4.2	11.8	88.2
10	94.6	5.4	12.1	87.9

y: years old; *: pre-determined cut-off WC value for each age and gender.

Figure 2 shows the graphic representation of the 50^th^ percentile values (cm) of the WC set in the current study along with the values obtained from publications of 12 different countries, for boys (A) and girls (B) aged from 6 to 10 years. We detected that six-year-old Brazilian boys had a WC similar to the Mexican boys, the highest of all countries. Seven- to nine-year-old Brazilian boys have WC values lower than those observed in Mexican and Indian boys, and at 10 the values were also lower than the USA boys. Six, seven and ten-year-old Brazilian girls had similar or slightly lower WC values than those detected in Mexican and Indian girls, and 8 and 9 year-old girls also had WC values lower than Mexican girls.

**Figure 2:** – *Comparison of children 50th WC percentiles curves between 13 different countries. A: Comparison between 6-10 years old boys; B: Comparison between 6-1- years old girls. C: Comparison between boys (C) and girls (D) WC 50th percentiles curves from different Brazilian studies.*

Moreover, Figure 2 presents the 50% percentile of WC values of the Brazilian boys (C) and girls (D) and in a previous study published in 2007. It may be seen that the current WC 50^th^percentile curves for Brazilian boys was higher than the values of 2007. The current WC 50^th^percentile curves for Brazilian girls are much higher than the values obtained in 2007, around 2.0 cm at 7 and 8, 2.5 cm at 9 years old, and reaching 4.0 cm at 10 years old.

Discussion

This study presents age and gender specific WC percentile values for a large and representative sample of Brazilian children aged 6-10 years, based on a multicenter longitudinal anthropometric evaluation of school children. Furthermore, it is the first study to propose that WC cut-offs values are associated with obesity, according to BMI for boys and girls from 6 to ten years old. Besides, our study demonstrated that in this school population the prevalence of fat excess was around 30%, with 15% boys and girls overweight and another 15% already obese. Indeed, these findings corroborate previous data pointing out that childhood obesity is an increasingly serious health issue nationwide and worldwide.^42-44^

These data complement the existing set of WC reference values obtained in some other countries and enhance the assessment capabilities of childhood obesity, in the most diverse sites for children care. Since several relationships may be established with the values of waist circumference, such as intra-abdominal fat deposition and cardiovascular disease risk factors in children,^45-48^ waist circumference could be adopted as an alternative or additional measurement to BMI in children. The strong correlation found between circumference values and BMI in this study demonstrates that such replacement or its additional use is feasible.

Furthermore, the proposed WC cut-off values for obesity provide a strong approximation of both normal weight (eutrophic) and obesity as defined by the international BMI categorization. No more than 7% of eutrophic children would have a higher WC cut-off value, which would indicate increased BMI. In such cases, other potential explanations may be present, such as initial fat excess caracterized by central obesity or additional clinical alterations (such as gastrintestinal disorders, amongst others). Regarding obese children, almost 87% may have been dignosed with obesity merely by measuring the WC. Moreover, WC is both simple and feasible measurement tool requiring inexpensive and user-friendly equipment, and little technical expertise, making it possible to carry out measurements on a scheduled basis in all schools. Our results demonstrate that Brazilian children present elevated 50^th^percentile WC values, with boys’ values occupying roughly the third top place and girls the forth top place of all countries analyzed. Even more importantly, plotting the 50^th^percentile WC values obtained in 2007 with the current ones confirms the increment of values across all ages in boys and more intensively in girls.

While The International Diabetes Federation (IDF) suggests that the metabolic syndrome (MetS) should not be diagnosed in children younger than 10 years, weight reduction should be considered in those with abdominal obesity, as measured by waist circumference. The correlation of visceral adipose tissue and waist circumference in children was confirmed and it is an independent predictor of insulin resistance, lipid levels and blood pressure - all components of MetS.

The IDF consensus definition of MetS in children and adolescents was intended to agree upon a universally accepted characterization for facilitating MetS diagnosis and to accelerate preventive measures before the child or adolescent develops diabetes or cardiovascular disease. Obesity, particularly in the abdominal region, is associated with increased risk for cardiovascular disease and type 2 diabetes.^49^

The present study circumscribed an area of 12 cities in the São Paulo State. We believe it may be representative of school children of a large area of Brazil, since it included the most significant number of children of private and governmental schools ever reported in Brazil. Moreover, São Paulo state has a high degree of miscegenation, and its countryside has an economic and social development comparable to the South and Southern Regions and even with many closer areas of the Central-West. As emphasized by several reports, the evaluation of cardiometabolic disorders in children is only feasible when specific references to the association between age, gender and ethnic origin and health risks are available. With a sample of 9,713 subjects from 2 to 18 years old, including 3,414 African-American, 2,746 European-American, and 3,553 Mexican-American (MA), Fernandez et al.^14^have described and provided estimates of the distribution of WC percentiles curves widely used in different countries, the well-known NHANES data. On the 75^th^ and 90^th^ percentiles, MA girls showed the fastest overall increase among all girls. At any of the percentiles considered, MA persons showed the highest overall WC and the fastest overall rate of WC increase with age. This data supports the high WC percentiles among Brazilian children and the most significant increase among Brazilian girls.

Based on the robust data they collected, Fernandez et al.^14^stressed that careful attention should be concentrated to children whose WC values fall in the 75^th^ and 90^th^percentile, since this drop help identify children at risk for various comorbidities and they strongly suggest prevention actions [against these situations]. All percentiles values found in our study were higher than those described by Fernandez et al.^14^ Among German children (6-10-year-old boys and girls), the 97^th^WC percentile was associated with abdominal obesity.^50^

Healthy lifestyle habits, including engaging in a healthy diet and physical activity, may lower the risk of becoming obese and developing related diseases.^51,52^ The dietary and physical activity behaviors of children and adolescents are influenced by many societal environments, including families, communities, schools, child care settings, medical care providers, faith-based institutions, government agencies, the media, and the food and beverage industries and entertainment businesses.^53-56^ Schools play a particularly critical role in establishing a safe and supportive environment with policies and practices that support healthy behaviors. They also provide opportunities for students to learn about and practice healthy eating and physical activity behaviors.^56^ Therefore, the importance of the present study was to provide representative values of WC of our children that may be used as an assessment tool to help meet public health recommendations.

Another significant contribution in the development of national epidemiological data is the Study of Cardiovascular Risks in Adolescents - (Portuguese acronym "ERICA" - Estudo de Riscos Cardiovasculares em Adolescentes).^57^ It is a large cross-sectional study at the national and school-based levels and a pioneering study that aimed to assess the prevalence of cardiovascular risk factors, including metabolic syndrome components in approximately 85,000 students, aged 12 to 17 years. A recent publication of ERICA described that the prevalence of metabolic syndrome was around 2,6% and that the most common combinations of elements, referring to 3/4 of combinations, were: enlarged waist circumference (WC), low HDL-cholesterol (HDL-c) and high blood pressure; followed by enlarged WC, low HDL-c and high triglycerides; and enlarged WC, low HDL-c, high triglycerides, and blood pressure. Therefore, the results of ERICA reinforce the importance of WC as a potential indication of a more global disarrangement that could determine the metabolic syndrome.

The main limitation of the present study is the described percentile curves based on a sample of children from only one state, São Paulo, and not derivated from randomly selected regions across Brazil. This fact may restrict the generalization of our results to children across the entire country. Furthermore, to validate the cut-off points for overweight and obesity, it is necessary to test the respective values in a different coorte of children.

## Conclusion

Age and gender specific reference curves of WC for Brazilian children and cut-off values for obesity risk may be used for national screening and interventional studies to reduce the obesity burden in Brazil.
